# Deep-Learning-Based Stroke Screening Using Skeleton Data from Neurological Examination Videos

**DOI:** 10.3390/jpm12101691

**Published:** 2022-10-11

**Authors:** Taeho Lee, Eun-Tae Jeon, Jin-Man Jung, Minsik Lee

**Affiliations:** 1 Department of Electrical and Electronic Engineering, Hanyang University, 55 Hanyangdaehak-ro, Sangnok-gu, Ansan 15588, Korea; 2 Department of Radiology, SMG-SNU Boramae Medical Center, Seoul National University College of Medicine, Seoul 07061, Korea; 3 Department of Neurology, Korea University Ansan Hospital, Ansan 15355, Korea; 4 Zebrafish Translational Medical Research Center, Korea University, Ansan 15328, Korea

**Keywords:** stroke diagnosis, landmark extraction, recurrence plot, deep learning

## Abstract

According to the Korea Institute for Health and Social Affairs, in 2017, the elderly, aged 65 or older, had an average of 2.7 chronic diseases per person. The concern for the medical welfare of the elderly is increasing due to a low birth rate, an aging population, and the lack of medical personnel. The demand for services that take user age, cognitive capacity, and difficulty into account is rising. As a result, there is an increased demand for smart healthcare systems that can lower hospital admissions and offer patients individualized care. This has motivated us to develop an AI system that can easily screen and manage neurological diseases through videos. As neurological diseases can be diagnosed by visual analysis to some extent, in this study, we set out to estimate the possibility of a person having a neurological disease from videos. Among neurological diseases, we focus on stroke because it is a common condition in the elderly population and results in high mortality and morbidity worldwide. The proposed method consists of three steps: (1) transforming neurological examination videos into landmark data, (2) converting the landmark data into recurrence plots, and (3) estimating the possibility of a stroke using deep neural networks. Major features, such as the hand, face, pupil, and body movements of a person are extracted from test videos taken under several neurological examination protocols using deep-learning-based landmark extractors. Sequences of these landmark data are then converted into recurrence plots, which can be interpreted as images. These images can be fed into convolutional neural networks to classify stroke using feature-fusion techniques. A case study of the application of a disease screening test to assess the capability of the proposed method is presented.

## 1. Introduction

The prevalence of neurological disease increases with age. Accordingly, both related medical costs and personal and social burdens increase. Therefore, in a super-aged society, such as Korea, the personal, social, and national interests in the prevention, initial diagnosis, and treatment of neurological diseases in the elderly are gradually increasing. Typical neurological diseases in the elderly include stroke, dementia, and Parkinson’s disease.

Stroke is a clinical syndrome of cerebral defects that lasts for more than 24 h and has no obvious features other than vascular features [1]. According to the global burden of disease study in 2015, neurological disorders, including stroke, accounted for 16.8% of all deaths [2,3]. The risk of stroke generally increases in people over 60 years of age as blood vessels become harder and narrower with aging. In the United States, stroke is the fifth leading cause of death, generating health care costs of up to USD 33.6 billion each year [4]. In Korea, the nationwide cost for stroke care was KRW 3737 billion (USD 3.3 billion) in 2005 [5]. Disease management is highly dependent on prompt diagnosis, particularly for ischemic stroke. According to current ischemic stroke guidelines, patients are eligible for intravenous thrombolysis up to 4.5 h from symptom onset and endovascular thrombectomy without advanced imaging within 6 h of symptom onset [6,7,8]. A prompt diagnosis with a suitable treatment decision is crucial for acute stroke management. Pre-screening high-risk stroke patients can help deal with these emergencies.

As the average age of the population increases, and the number of medical personnel becomes insufficient, the need for an automatic diagnosis system, or a system that can aid in medical procedures, increases. Early screening and diagnosis of neurological diseases, including stroke, are mainly based on neurological examinations by experts in the neurological field. The accessibility of special neurologists or neurological clinics for the elderly population is declining. Accordingly, researchers have been striving to identify automatic detection methods for neurological disorders using machine intelligence. For example, some researchers have focused on assessing facial paralysis through studies using facial landmarks [9,10], 3D information [11,12], and optical flow [13].

With the remarkable success of deep learning in computer vision problems, such as segmentation, detection, classification, etc., many researchers are developing diagnostics systems based on deep neural networks. Predicting lung cancer treatment response by analyzing time-series CT images of patients [14] or identifying pneumonia and COVID-19 using X-ray pulmonary images [15] are examples. In contrast, a deep-learning-based approach, using a one-dimensional convolutional neural network (CNN), has significantly improved the ability to convert brain signals into clinically useful features for epilepsy diagnoses [16]. A deep-learning-based automatic diagnosis system can significantly reduce the time and expense of diagnosis; however, it requires large amounts of medical data for training. Therefore, these methods face challenges in data collection.

In this study, we propose a new deep-learning-based approach to analyze the presence of a stroke in a patient where it is suspected or where there is considerable risk of its occurrence. We formulate this task as a binary classification problem, that is, stroke (or related disorders) vs. normal. The input data for the proposed method are videos, which were recorded while patients performed a certain set of motions according to predefined test protocols. These are captured by ordinary video cameras and there is no need for expensive devices such as CT or MRI scanners. A straightforward approach to process these videos is to use a video-based deep model; however, this is computationally quite expensive. The goal of this paper is to propose a stroke screening method that can be used in public, so feeding videos into a neural network directly is not a suitable approach. To resolve this issue, in this paper, the videos go through deep-learning-based human landmark detectors to extract the time series of the major landmarks. Then, these time-series data are fed into deep neural networks to estimate the possibility of a stroke.

The contributions of this paper can be summarized as follows:

1. To the best of our knowledge, the proposed deep-learning-based approach is the first attempt to estimate the presence of a stroke using major human landmarks. Here, the proposed method does not focus on a specific part of a person, but on various parts, such as the face, pupils, body, and hands, to make a more robust decision. The data from actual patients were used to train and validate the proposed method.

2. A novel variant of the recurrence plot transform is proposed to efficiently process a vast amount of the landmark data extracted from the videos.

3. Pretrained weights, trained separately on each neurological protocol, were used for training the proposed network to improve its final performance. This was inspired by the observation that mildly affected patients can exhibit normal behaviors in many individual neurological examination protocols.

We performed extensive experiments under various different settings of the proposed method and reported the area under the curve (AUC) values of receiver operating characteristic (ROC) curves for the screening test set. The proposed method can potentially be extended to other clinical tests, especially for neurological disorders that result in muscular motion abnormalities.

The remainder of this paper is organized as follows: Related work is introduced in Section 2. The KUAH dataset and the proposed method are described in Section 3. The experimental results are presented in Section 4. We conclude the paper with a discussion in Section 5.

## 2. Related Works

### 2.1. Stroke Diagnosis Based on Machine Learning

Stroke is a common but fatal vascular disease. It is the second most common cause of death and the third major cause of disability [17]. The gold standard test for stroke diagnosis is a brain screening MRI or CT scan to detect brain lesions. In addition, there are two commonly adopted clinical tests, the Cincinnati Pre-hospital Stroke Scale (CPSS) [18] and the face arm speech test (FAST) [19]. Both methods assess the presence of unilateral facial droop, arm drift, and speech disorders. The patient is requested to repeat a specific sentence (CPSS) or to have a conversation with the doctor (FAST), and abnormality is disgnosed when the patient slurs, fails to organize their speech, or is unable to speak.

Machine learning (ML) has been applied in medicine increasingly frequently over the past decade. In the case of strokes, machine learning algorithms have already been used in clinical applications for automatic diagnosis. ML methods have been applied to the diagnosis of a stroke [20], prediction of stroke symptom onset [21,22], assessment of stroke severity [23], analysis of cerebral edema [24], and outcome prediction [25]. The data used in these studies were composed of medical records, CT images, or MRI scans. Such data forms are quite expensive; therefore, they cannot be collected easily in large quantities. Moreover, there has been a rapid increase in deep learning for image-based stroke diagnosis [26,27], where a large amount of training data are required. This approach can provide better results because of the power of deep learning. However, the cost of data collection is several times greater. Another downside of the above approaches is that they are all based on images; they often fail to capture temporal changes in human muscular motions which can be especially useful for diagnosing a stroke.

### 2.2. Recurrence Plots

A recurrence plot (RP) [28] is a visualization method that transforms the trajectory of an *m*-dimensional phase space in two dimensions. Equation (1) is a popular method for generating an RP.

(1) $$R\left(i,j\right)=\left\{\begin{array}{cc} 1,if∥\begin{array}{c} \vec{x}\left(i\right)-\vec{x}\left(j\right)\leq \varepsilon \end{array}∥,\; & \\ 0,otherwise. \end{array}\right.$$

In (1), *R* is a matrix where the (i,j)-th element is set to 1 if $\vec{x}\left(i\right)-\vec{x}\left(j\right)$ is less than a given threshold ϵ. We should select ϵ carefully since an excessively small ϵ makes the RP sensitive to noise and an excessively large ϵ can make an RP contain trivial information. An RP with a well-chosen ϵ provides information about the temporal correlation.

**Figure 1:**
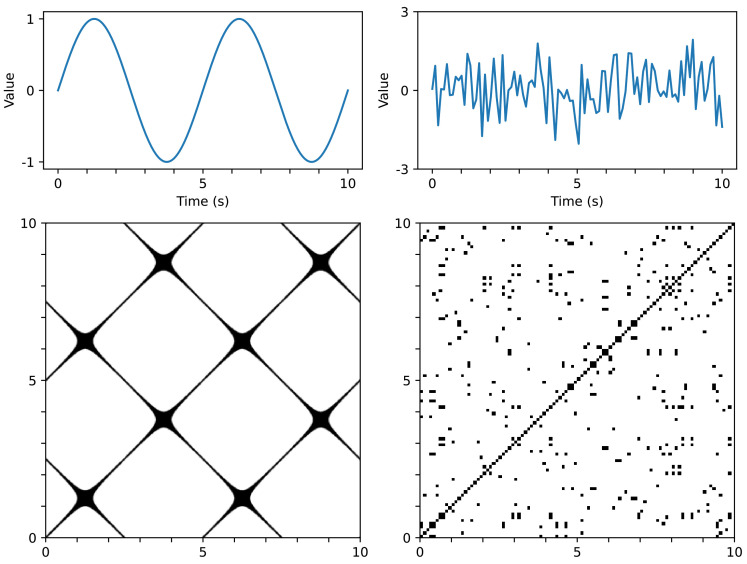
Examples of time-series data and the corresponding RPs.

We chose the threshold ϵ=80 to generate an RP in this study based on our empirical experience. Figure 1 shows examples of signal data and its RP. The image transformed by the RP algorithm depicts a collection of time pairs whose locations are the proximity of each other. In other words, the visual representation of the RP can provide information on time-series data and be efficiently utilized by a convolutional neural network (CNN) [29].

## 3. Materials and Methods

### 3.1. The KUAH Dataset

The clinical dataset for this thesis was acquired in Korea University Ansan Hospital (KUAH). The institutional review board (IRB) of KUAH approved the study protocol (2018AS0291).

Screening for subjects was performed for in-patients and out-patients who were admitted or visited the Department of Neurology at KUAH. Specifically, the control group was collected through a clinical trial advertisement at the hospital. The control group had no medical conditions or neurologic diseases that could be related to abnormal neurologic findings or impaired gait. Patients who provided informed consent were enrolled in the study. A large pool of patients diagnosed with stroke was screened. A total of 382 individuals were finally recruited to collect neurological examination videos from August 2017 to November 2020, according to predefined data collection protocols designed by a collaboration between KUAH and Hanyang University. The subjects were either stroke patients or healthy controls. The median and interquartile range (IQR) values of the participants’ age were 62 and 18, respectively. Among these participants, those in their 60 s to 70 s accounted for the majority (46%), followed by those in their 40 s and 50 s (40%). The percentage of men was higher than that of women.

The stroke diagnosis was confirmed by a stroke neurologist (J., J.-M.) through clinical and radiological evidence. In this study, we focused on a binary classification task to distinguish stroke cases from normal subjects, regardless of the stroke subtypes, including ischemic and hemorrhagic strokes. Thus, of 382 participants, 259 were labeled “stroke” and the rest “normal”.

Each subject was asked to perform seven tasks: (1) movement of the extraocular muscles; (2) making a frowning face; (3) repeating sentences; (4) raising hands side-by-side forward; (5) finger tab exercise; (6) pointing at own nose with their finger and then touching an installed object repeatedly; and (7) gait. The ability to perform correct facial expressions and body movements is an important indicator of stroke; that is, if the subject slurs, mumbles, limps, or has difficulty in enacting body motions, they have a high chance of stroke [18,19]. The subjects were asked to sit in a chair for most of the tasks, except for the gait task. The protocols corresponding to these seven tasks were named extraocular movement (EOM), facial palsy, dysarthria/aphasia, weakness of the upper extremities, finger tab, ataxia of the upper extremities, and gait, respectively.

The subjects were video-recorded as they performed the above seven tasks. Six tasks were recorded with four full-HD cameras from the upper, middle, left, and right directions, respectively, and the gait task was recorded with one frontal camera.

**Figure 2:**
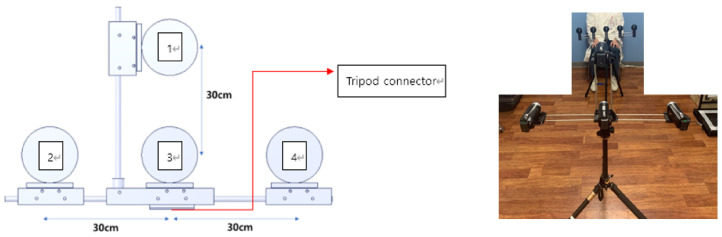
Camera station used for collecting the KUAH Dataset.

Figure 2 shows a picture depicting the camera station used for collecting the KUAH dataset. Four Sony Handycam HDR-CX405 cameras were installed on the station as follows: The distances between Camera 3 and the others were 30 cm; Cameras 1, 2, and 4 were tilted in the direction of the subject at an angle of 15 degrees. The station was fixed on a one-meter-tall tripod.

Apart from the gait protocol, all the other protocols were recorded in one take. Therefore, they needed to be separated into individual videos afterwards. To facilitate this process, a screen fence with a distinctive color was used to differentiate the protocols; that is, it appeared in between every two consecutive tasks (or protocols). The videos were separated automatically if there was a sudden change in the distribution of colors in the frames; all the results were verified manually.

Each video had a label and metadata information on clinical impressions by a stroke neurologist (J., J.-M.) (indicating the expert’s judgement on whether the subject had performed the task normally or not and the corresponding symptom if the task was not normally performed) and each subject had a ground truth label of whether they had a stroke and another label for its severity. In the proposed method, the label for each video was used in pretraining, while that for each subject was used in the main training procedure.

The KUAH dataset provides real-world data from stroke patients as well as from normal controls, which makes the proposed method useful for practical clinical use, for example, to assist in the remote diagnosis of strokes. A distinctive characteristic of the KUAH dataset is that it provides various examination protocols, which focus on many different parts of the human body and are comprehensively recorded using four cameras. Unlike other studies that required more expensive data forms, we only used RGB videos, which can reduce the overall cost of diagnosis.

### 3.2. Proposed Method

**Figure 3:**
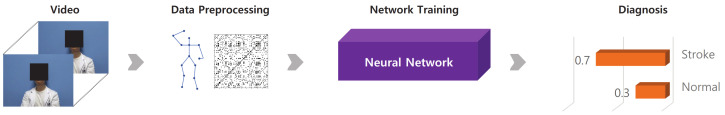
The overall workflow of the proposed algorithm.

In this study, we propose a deep-learning-based stroke diagnosis method. This section introduces the data preprocessing methods, the proposed classification network, and the detailed modules and techniques used in the network architecture. The overall procedure of the proposed method is shown in Figure 3.

The overall workflow of the proposed algorithm consists of four steps: (1) collecting neurological examination videos, (2) processing the videos using human landmark detectors and converting the results to RPs, (3) feeding the RPs into the proposed network, and (4) estimating the possibility of a stroke.

#### 3.2.1. Landmark Extraction

In our task, we needed pose, hand, face, and pupil landmarks from neurological examination videos to train the neural network. There are many methods to extract human landmarks in an image or video but, recently, deep-learning-based approaches have exhibited the best performance in landmark detection. Accordingly, we used deep-learning-based methods in this paper.

The details of landmark extraction for each part are as follows:

**Pose:** There are two popular approaches to multi-human pose estimation. First, OpenPose [30] is a bottom-up approach in which networks first detect the body parts or keypoints in an image and then map proper keypoints to form pairs and calculate the final poses. Alternatively, AlphaPose [31] is a top-down approach that attempts to extract a more accurate region from an unclear bounding box by adding a symmetric spatial transformer network. Because AlphaPose reported better performance than OpenPose [30], in this study, we chose AlphaPose to estimate body joints.

The estimated pose consists of 18 body joints: Lear (left ear; the following names are represented in a similar manner), Leye, Reye, Lshoulder, Rshoulder, Rear, Nose, Neck, Lwrist, Rwrist, Lhip, Rhip, Lelbow, Relbow, Lknee, Rknee, Lankle, and Rankle.

**Hand:** We extracted 2D hand skeletons using a hand keypoint detection model proposed in [32], which produces keypoints for both the right and left hands. The outputs of the model were 21 keypoints, four points on each finger and one point on the wrist. This model uses a training process called multiview bootstrapping with multiple cameras, to produce a fine-grained detector for hand keypoints with greater robustness to noise and occlusion. This model was selected in this study because it achieved a performance comparable to methods based on depth sensors. OpenPose already contains a good implementation of this hand pose estimation model [30]; therefore, we adopted this in our framework.

This model can produce precise hand keypoints that are crucial to representing fine hand movements, but there is one problem: to detect the hand keypoints, we need to know where the hand bounding box is in the image. Accordingly, we should either find this by a separate hand detection model or speculate on the location based on the result of a body pose estimator. In this study, we chose the latter approach, in which the pipeline is described as follows:

First, body keypoints were extracted using a body-pose estimator in the corresponding frame using AlphaPose.

Second, we cropped the hand image from the entire image using the wrist joints. Here, the location and size of the hand patch were set as follows: The estimated center of the patch was set to the extrapolated point of the wrist and corresponding elbow (20% farther point from the wrist); the length of the square patch was set to 1.2 times the distance between the two shoulders. The cropped image was then resized to a 368 × 368 resolution.

Finally, the hand keypoint extraction model was applied to extract the hand keypoints and their coordinates were restored to the original image’s coordinate system.

**Figure 4:**
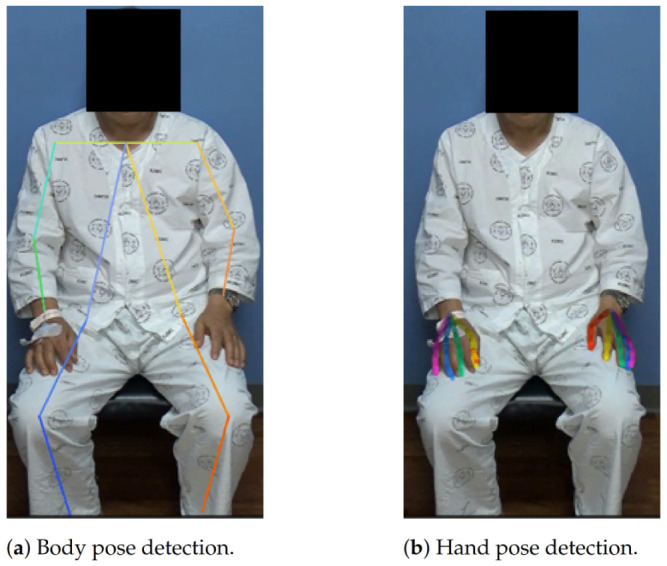
The example results of (**a**) AlphaPose [31] and (**b**) hand pose detection in OpenPose [30,32]. The estimated body pose consists of 18 joints and the estimated hand consists of 21 keypoints on each hand.

This pipeline cannot detect a hand that is not bound to any of the body keypoints. This might be avoided by using a separate hand detection model, but we found empirically that the general accuracy was much better when using speculated locations based on the body-pose estimator. The example results of the AlphaPose model and hand keypoint extraction pipeline are shown in Figure 4.

**Face:** RetinaFace [33] is a one-stage face detection model that utilizes multi-task learning. This algorithm exhibited excellent performance on the WIDER Face dataset [34]. Hence, we chose this model to obtain face bounding boxes. Style aggregation network (SAN) [35] is a heatmap-based approach based on the modified ResNet-152 backbone [29]. This algorithm shows satisfactory performance for both the 300 W [36] and ALFW [37] datasets. Hence, we selected this model for facial landmark detection. In summary, our facial landmark detection pipeline is as follows: First, the input image is fed into the RetinaFace model to obtain a face bounding box. Second, a cropped image from the bounding box is used in the SAN model to retrieve the facial landmarks.

**Pupil:** Many gaze estimation models estimate the location of a pupil, and we chose GazeML [38], which is a high-performance gaze estimation framework based on deep learning. GazeML utilizes an intermediate 2D representation instead of directly estimating the pitch and yaw of the eyeball to improve its performance. In our framework, the face landmarks explained above were utilized to roughly locate the eye positions, for which the cropped patches were fed into GazeML to extract the pupil landmarks.

#### 3.2.2. *N*-Divided Recurrence Plots

RP is a method of representing time series data using a 2D image based on distances between data points. In this study, the input data are the trajectories of various human landmarks, which are a set of time-series data. Hence, RP can be effectively utilized for this data.

Each keypoint that changes over time should be converted into a recurrence plot. However, the input videos in this study have a significant length in terms of time, and converting the landmark trajectories to corresponding RPs can lead to large image sizes. To resolve this issue, we propose a new technique for utilizing RP in deep learning; that is, dividing the time series into several sections, converting them individually to RP, and then concatenating them along the channel dimension to form a smaller image with *N* channels. This technique will be referred to as *N*-divided RP hereafter.

**Figure 5:**
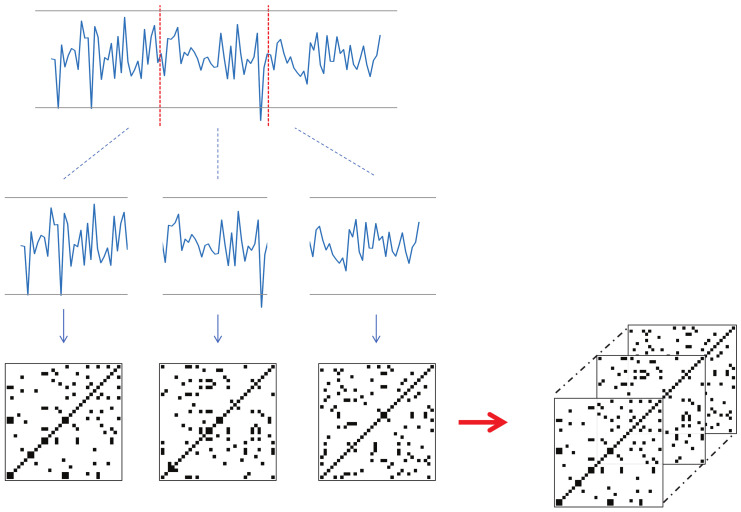
An example of three-divided RP. The entire sequence is divided into three parts, where each part is converted to an RP. The resulting RPs are concatenated to form a three-channel image.

If we transform time-series data to RP for the entire time *T*, the required memory complexity will be $O(T^{2})$, whereas an *N*-divided RP can express the entire time-series dataset with only $O(\frac{T^{2}}{N})$ (*N* pieces of $O({\left(\frac{T}{N}\right)}^{2})$). Although the *N*-divided RP excludes long-range relationships in its representation, most of the meaningful indicators of a stroke are concentrated in local regions, so this input representation is sufficient for the proposed method. Figure 5 shows an example of a three-divided RP. Here, the input time-series data had 68 channels, and the length was 90. The resulting three-divided RP becomes a 30×30 image with 204 channels (68 channels multiplied by three).

**Figure 6:**
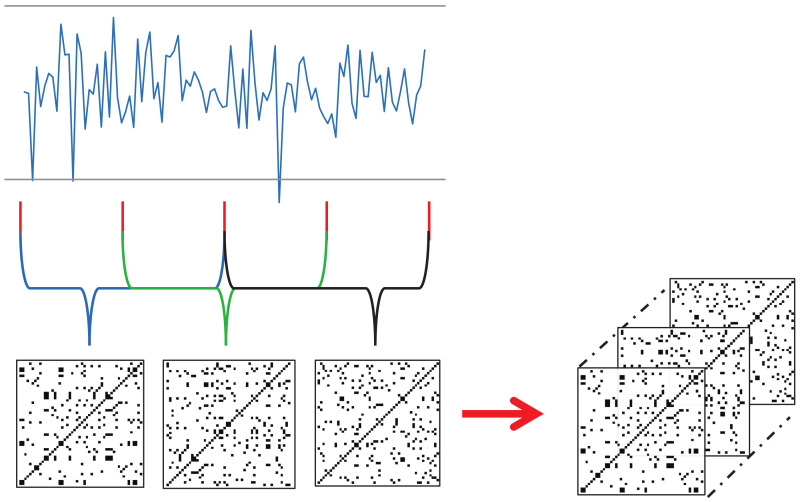
An example of overlapped three-divided RP. The sequence is divided with 50% overlaps between consecutive parts. The parts are converted to RPs and then concatenated to form a three-channel image.

One issue with the *N*-divided RP is that the regions near the borders of the sections may not receive enough attention. To resolve this issue, we created another version of *N*-divided RP: called overlapped *N*-divided RP, where consecutive sections overlap with each other by 50%. Figure 6 shows an example of the overlapped three-divided RP. The overlapped version produced a larger result than the original *N*-divided RP. For example, let us suppose that the length of the input sequence is 90, and *N* is three. Then, the size of the corresponding original RP will be 30×30, whereas that of the overlapped RP will be 45×45.

#### 3.2.3. Pretraining Subnetworks

**Figure 7:**
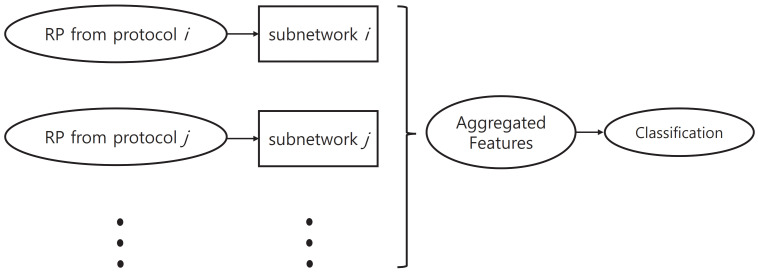
An outline of the proposed network structure.

In the proposed method, we used a network structure that consists of multiple subnetworks. These subnetworks were designed to learn protocol-specific features. Figure 7 shows an outline of network structure.

In the case of mild patients, it is quite common for them to perform normally in some of the neurological examination protocols. In these cases, the data for individual protocols were labeled “normal” in the KUAH dataset. These individual labels can be used in training the proposed method to improve its final performance. If we do not utilize these additional labels and train the entire network with only the final disease label end-to-end, then the training of each subnetwork will not be conducted efficiently. Accordingly, in this study, we first pretrained each subnetwork based on labels of the corresponding protocol, which was then attached to the entire network structure so that end-to-end training could be conducted.

ResNet [29] has demonstrated remarkable performance and is frequently described in the literature. Accordingly, we chose ResNet-18 as the base architecture which had the fewest trainable parameters among the ResNet architectures because the size of the training data was relatively small. This architecture was used for all subnetworks to estimate the results of the individual examination protocols. We also evaluated other backbone networks, such as ResNeXt [39] and EfficientNet [40]. ResNeXt adopts group convolution [41] in ResNet bottleneck blocks, which converts the multi-path structure into a unified operation. It achieved second place in the ImageNet Challenge [42]. EfficientNet, on the other hand, achieved high performance on the ImageNet dataset by making CNN much more effective, based on the observation that balancing network resolution, channel width, and depth can lead to better performance. The experimental results for the pretraining are presented in Section 4.

#### 3.2.4. Selective Dropout

As will be demonstrated later in the experimental analyses, the performance of the pre-trained subnetworks, as described above, shows some disparities. This indicates that different subnetworks may vary in their reliability in estimating the final answer. Accordingly, it is necessary to control the importance of the output features of different subnetworks when they are combined together at the final stage of the proposed network. To address this issue, we propose a new technique called selective dropout to boost the final performance of the proposed network. The basic process of selective dropout is described below.

**Figure 8:**
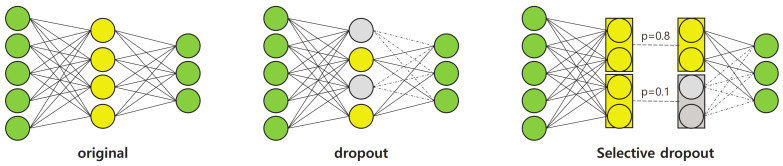
An example of selective dropout. (**Left**): no dropout, (**middle**): the original dropout [43], (**right**): selective dropout.

Selective dropout is a combination of several dropout modules which have different probabilities. Dropout [43] is a technique used to regularize a neural network by stochastically dropping features. In the proposed selective dropout, each dropout module is connected to each subnetwork, and the probability of dropout is set according to the importance of the subnetwork. Here, the probabilities are predefined by the users. As shown in Figure 8, the selective dropout drops features in different paths with different frequencies, unlike the original dropout. This can control the impact of different subnetworks on the final decision. The experiments described in Section 4.7 involve a comparison between different dropout techniques as well as a simpler weighting strategy, which demonstrates that selective dropout exhibits the controlling effect.

#### 3.2.5. Overall Network Structure

**Figure 9:**
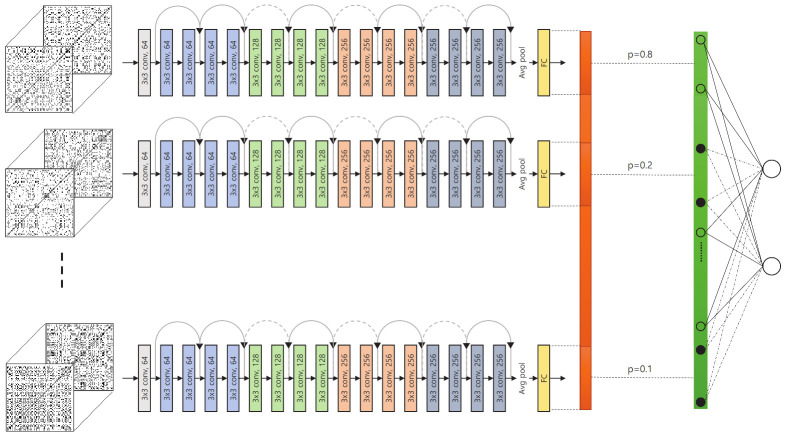
Proposed network structure with selective dropout.

The overall structure of the proposed network is shown in Figure 9.

With a set of *N*-divided RPs from the input landmark data, we first extracted features via the corresponding subnetworks for individual examination protocols. The features extracted from the subnetworks were concatenated into a single long vector; this final feature vector went through selective dropout and a fully connected layer to yield the estimated diagnosis. This structure is designed to enable subnetworks to learn protocol-specific features to improve the overall performance.

Because the proposed method is a classification problem, we used cross-entropy loss to guide the proposed network. In general, a large amount of data is required to train a deep neural network. However, the size of the KUAH dataset used in this study is relatively small. One of the techniques we used to overcome this problem is the pretraining of subnetworks, as explained in Section 3.2.3. To help address this problem, we also utilized individual protocol labels in the fine-tuning procedure. This formulates the training procedure as a multi-task learning problem; that is, the proposed network must estimate the final answer (whether the subject has a stroke), as well as evaluate the individual protocols (whether the subject performed each task successfully or not). This can provide further guidance to the proposed network and stabilize the overall training procedure. Accordingly, the overall loss function for the proposed method is given by (2).

(2) $$L=l_{stroke}+\sum_{i}^{}\lambda_{i}l_{protocol,i}$$

where $l_{stroke}$ is the loss for the final label, $l_{protocol,i}$ is the loss of the *i*-th examination protocol, and $\lambda_{i}$ is the corresponding weight.

## 4. Results and Discussion

### 4.1. Detailed Settings of the Training Procedure

In this paper, the proposed algorithm was implemented with PyTorch [44] and evaluated on a single NVIDIA TITAN Xp GPU throughout the experiments. For all the experiments, training, including pre-training and fine-tuning, was conducted for 50 epochs with a mini-batch size of one using the Adam optimizer ($\beta_{1}=0.9,\beta_{2}=0.999$) and no weight decay was used unless otherwise stated. The learning rates were set to $10^{-3}$ for pre-training and $10^{-4}$ for fine-tuning, unless otherwise stated. For the evaluation, we reported the area under the ROC curve (AUC). All the hyperparameters explained here were configured manually according to a five-fold cross-validation, except in Table 1, where only a single (pre-)training procedure was conducted for each subnetwork.

In most of the experiments, an overlapped *N*-divided RP was used, except for Section 4.4. Since the usual lengths (time) of the protocols are different, we set *N* of the overlapped *N*-divided RP differently for different protocols, such that the resulting image size approached 224×224, which was the input size of the backbone models. The width and height of the resulting images were not exactly 224; therefore, we resized them to 224×224 using bilinear interpolation. For example, in the EOM test, the average number of total frames in a video was approximately 1500; therefore, we set N=9. Similarly, we set *N* to 3, 3, 5, 5, 7, and 15 for the remaining tests, respectively. Note that we removed the frames that corresponded to the remainder after division by *N*.

For most of the experiments, we used ResNet-18 [29] as the backbone model for the subnetworks. For some experiments, we also tested three other backbone networks: ResNet-34, ResNeXt-50 [39], and EfficientNet-B2 [40] were used to observe the effects of the different backbones. We adjusted the initial convolution kernel sizes of these backbones according to the channels of the overlapped *N*-divided RP and the output channels of the final fully connected layers were set to two (stroke vs. normal).

When retrieving keypoints using landmark detectors, the coordinates were set to zero if they failed to find the correct landmarks. For different protocols, different landmarks were used. For example, because the EOM test is about facial expression, we used only the face and pupil coordinates, which included 70 landmarks. The same landmarks were used for the facial palsy and dysarthria/aphasia tests. By contrast, 60 landmarks from the body and hands were used for the weakness, finger tab, and ataxia tests. Lastly, 18 body landmarks were used for the gait test.

Unless otherwise stated, we used two simple augmentation techniques for the landmark sequences, i.e., scaling and jittering. For jittering, random noise from a Gaussian distribution, with a mean μ=0 and a standard deviation σ=0.03, was added to the sequences. For scaling, the magnitude of all elements in the time series was increased or decreased by a scalar determined by a Gaussian distribution, with μ=1 and σ=0.1.

In the training procedure, the losses in (2) and selective dropout were used, unless otherwise stated. The protocol-specific losses were scaled by weights which were the same as the corresponding probability values of the selective dropout. These weights (or probabilities) were set so that the overall network focused on protocols, where the subnetworks showed higher performance in pre-training because the features from these were likely to be more reliable. Accordingly, the weights were set to 0.3, 0.3, 0.3, 0.9, 0.8, 0.7, and 0.7, respectively, for the seven protocols (see Section 4.3 for more details).

### 4.2. Data Configurations

**Figure 10:**
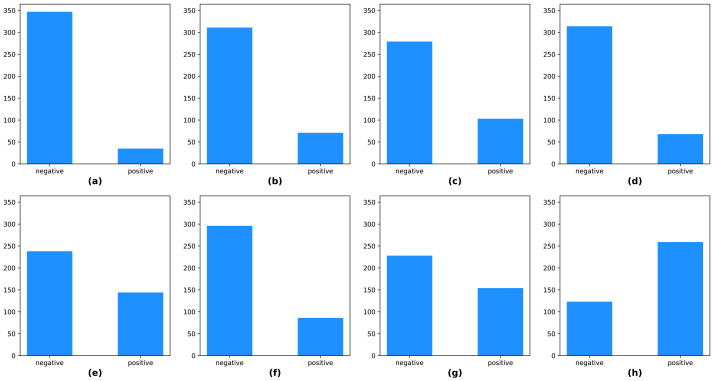
Label distributions of (**a**) EOM, (**b**) facial palsy, (**c**) dysarthria/aphasia, (**d**) weakness, (**e**) finger tab, (**f**) ataxia, (**g**) gait, (**h**) stroke.

The KUAH dataset was acquired from 382 individuals and split into two subsets, approximately 70% for training, and 30% for testing. The training set had 266 samples, and the test set had 116 samples. There were class-imbalances in the labels, including the protocol labels and final labels of the KUAH dataset, as shown in Figure 10. Hence, we carefully divided the subjects into training and test sets so that the labels were as evenly distributed as possible.

### 4.3. Analysis of Pre-Training Results

In this section, we discuss the pre-trained subnetworks introduced in Section 3.2.3. In Table 1, the subnetworks were pre-trained separately on individual neurological protocol tests. The performance reported here represents the AUC value for the corresponding protocol tests.

**Figure 11:**
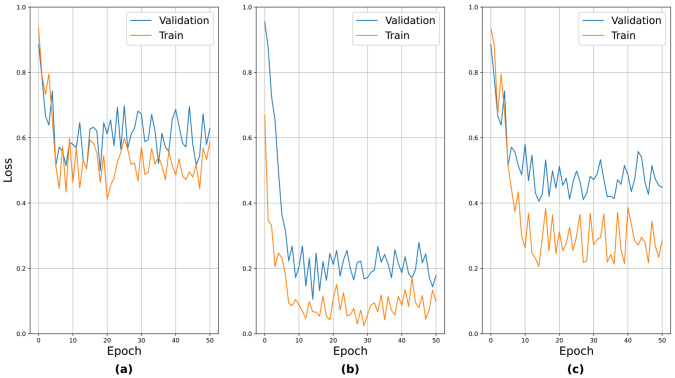
Learning curves in pre-training for (**a**) EOM, (**b**) Weakness, and (**c**) Ataxia.

The results for the EOM, facial palsy, and dysarthria/aphasia protocols, which involved facial expressions, were worse than those involving body movements. We conjecture that the facial landmark detector had learned only on the faces of normal persons in public benchmark datasets, so it was difficult for it to capture the anomalies in the faces of patients with strokes. This observation was the basis of the idea behind the selective dropout proposed in Section 3.2.4. To verify the validity of the pre-training, the learning curves for the pre-training are presented in Figure 11. The above seven protocols can be roughly divided into three groups based on their relative performance: (i) those with relatively poor performance, i.e., EOM, facial palsy, and dysarthria/aphasia, (ii) those with relatively good performance, i.e., weakness and finger tab, and (iii) those with moderate performance, i.e., ataxia and gait. We sampled one protocol from each group and presented the learning curves. It can be seen that the curves generally showed stable convergence. An interesting observation was that the loss values after convergence reflected their actual performance, i.e., EOM had the highest loss value, while weakness had the lowest.

Table 2 shows the results of the final stroke screening tests depending on different pre-training methods. For this experiment, the network structure explained in Section 3.2.5 and its subnetworks were initialized with various pre-trained subnetworks, including those described above. Here, “Random weights” indicates that no pre-training was used, and the entire network structure was trained from scratch. In this case, the Kaiming uniform initialization in PyTorch was used to configure the initial weights and the entire network structure was trained for 50 epochs at a learning rate of $10^{-3}$. The other two configurations, starting with “pre-trained with” indicates that the entire network structure was fine-tuned (according to the setting introduced in Section 4.1) using pre-trained subnetworks. Here, we also tested another external pre-training dataset, i.e., the NTU-RGB+D dataset [45]. The NTU-RGB+D dataset is an open dataset designed for skeleton-based action recognition problems. The format of this data (trajectories of human landmarks) is similar to that of our input data; therefore, this may be another suitable candidate for the pre-training of the proposed method. For this dataset, the subnetworks were pre-trained for 200 epochs with a mini-batch size of eight using a learning rate of $10^{-3}$.

For pre-training of the subnetworks on the NTU-RGB+D dataset, we used the same *N* for generating overlapped *N*-divided RP, as explained in Section 4.1, and the resulting images were resized to 224×224. However, there was a problem that the NTU-RGB+D dataset only had 25 joints in the human body. The number of points required for each protocol was usually larger than 25 (i.e., 60 or 70), except for the gait protocol, which required 18 points. In the case of the gait protocol, we excluded some joints to match the number. Here, the joints that were far from those of the body landmark detector were excluded. For the other protocols, we increased the number of points by linearly interpolating the trajectories of the connected joints (i.e., the limbs). Specifically, we divided the limbs equally to produce additional points and the number of divisions was set such that the resulting points satisfied the requirements. However, this number of divisions might not become a natural number; therefore, we divided the longer limbs more than the others. For example, suppose that we needed to divide the limbs into 3.5 equal parts to produce the required number of points. In this case, according to the reference shape provided in the NTU-RGB+D dataset, longer limbs were divided into four parts, and the rest were divided into three parts.

In Table 2, it is apparent that using pre-trained weights was better than using random initialized weights. Among those using pre-trained initializations, “pre-trained with protocols” showed better final performance. The subnetworks pre-trained with individual protocols were designed to learn protocol-specific features, so it was anticipated that using these would be better.

**Figure 12:**
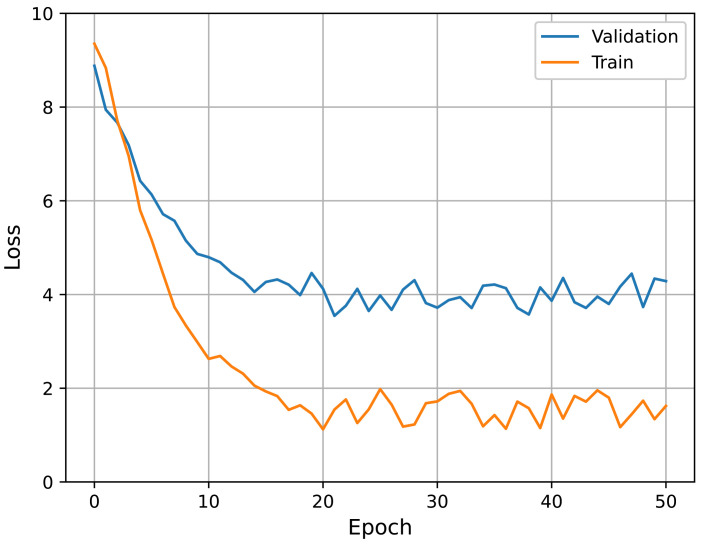
Learning curves in fine-tuning. Here, the subnetworks were pre-trained with individual protocols and the learning rate was $10^{-4}$.

We also performed an additional experiment for the choice of a fine-tuning method; that is, we tried different learning rates for fine-tuning. In Table 3, the term “freeze” implies that we froze the pre-trained subnetwork weights when fine-tuning the entire network structure. In this case, a learning rate of $10^{-3}$ was applied to train the additional layers (the final FC layers). In the other cases, all weights (including the subnetworks) were allowed to change according to the indicated learning rates. In the table, using a learning rate of $10^{-4}$ resulted in the best performance for both pre-training cases. In conclusion, the best configuration involved fine-tuning the overall structure with a learning rate of $10^{-4}$, using subnetworks pre-trained with the protocol labels. Accordingly, all experiments described hereafter follow this configuration. The learning curves of the best performing model (i.e., pre-trained with individual protocols and $Lr=10^{-4}$) in fine-tuning are presented in Figure 12. It can be seen that the curves show stable convergence.

### 4.4. Analysis on the Choice of Recurrence Plot Formats

To determine the effectiveness of the proposed overlapped *N*-divided RP, we compared it with its non-overlapping counterpart. In the case of *N*-divided RP, we used smaller *N* to produce an image with a similar size compared with the case of the overlapped version. Accordingly, we set *N* to 6, 2, 2, 3, 3, 5, and 10, respectively, depending on the protocols. The resulting images were resized to 224×224.

**Figure 13:**
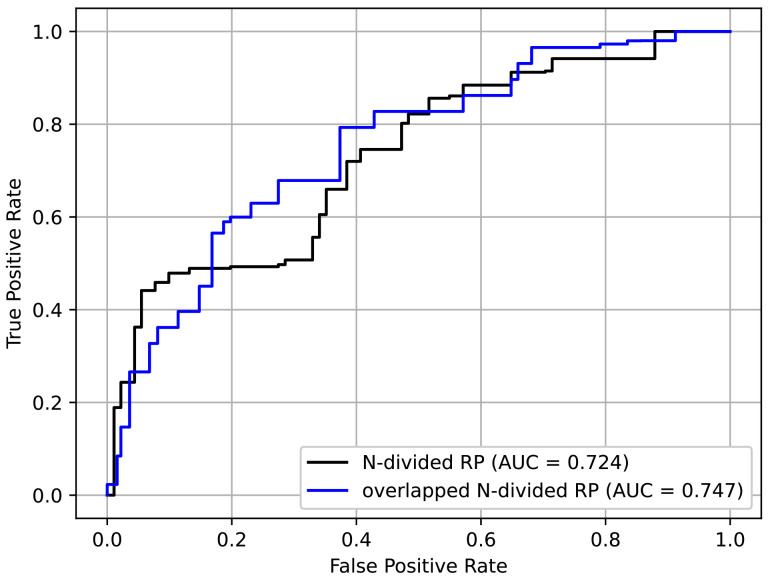
ROC curves for different RP formats.

In addition to the input format, all other settings followed those explained in Section 4.1 and the pre-training/fine-tuning method in Section 4.3. From Table 4, it is confirmed that the overlapped *N*-divided RP showed better performance than the original RP. This was because the overlapped *N*-divided RP can pay more attention to the regions near the borders of the sections than the *N*-divided RP. The ROC curves of the above experiment are shown in Figure 13. It can be seen that the overlapped *N*-divided RP showed better characteristics.

### 4.5. Analysis on Network Backbones

In this section, we discuss the network backbones introduced in Section 3.2.3. We experimented with several network backbones mentioned in Section 4.1.

**Figure 14:**
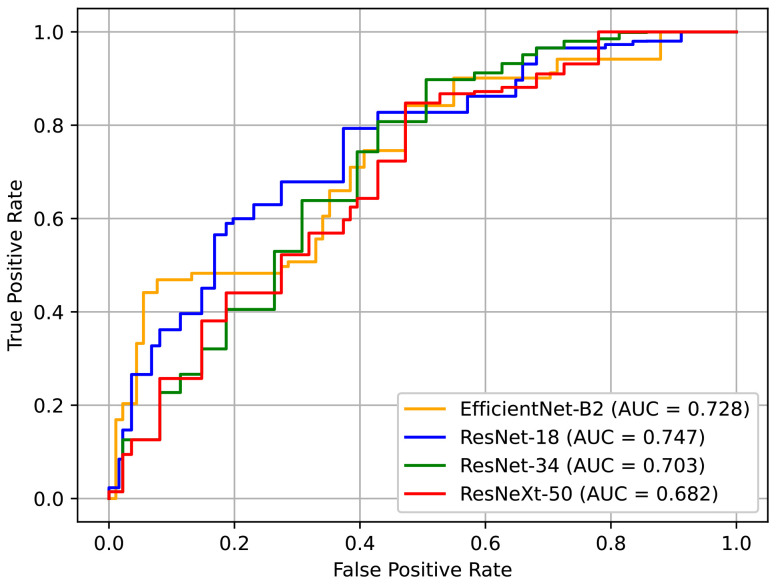
ROC curves for different network backbones.

Table 5 shows the performance of the networks based on different backbone architectures. In our case, the model with a smaller capacity was a better choice, especially because the dataset that we used was relatively small. The results showed that ResNet-18 and EfficientNet-B2 exhibited better performance, which supports this insight. ResNet-18 showed the best AUC of all the models. Although EfficientNet-B2 recorded the second-best performance, it represents a good alternative because it has less computational costs. Figure 14 depicts the ROC curves for these models. Here, it can be seen that the curve of ResNet-18 is generally more favorable.

### 4.6. Comparison to Non-RP-Based Baseline

**Figure 15:**
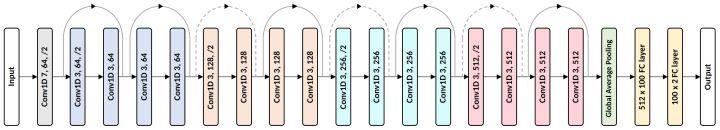
The architecture of 1D ResNet. “/2” in the conv blocks indicates that the stride was 2. A dotted skip connection means that the width and height are halved by an average pooling and the channel size is doubled by adding zeros.

In this section, we describe an additional experiment to compare the proposed method with a non-RP-based baseline using landmark data. We created the baseline by replacing the subnet backbones with 1D ResNet. The details of the 1D ResNet are shown in Figure 15. The channel sizes and the number of residual blocks were tuned to yield the best performance. Here, a residual block contains two sequences of 1D convolution, BN, and ReLU, and its input and output are connected with a skip connection. All the proposed techniques, i.e., the augmentation transform, selective dropout, and individual protocol labels, were used to provide a fair comparison. Moreover, even though we were not using RP for the baseline, we divided the entire sequence into *N* parts with overlaps to ensure that the input setting was as close as possible.

**Figure 16:**
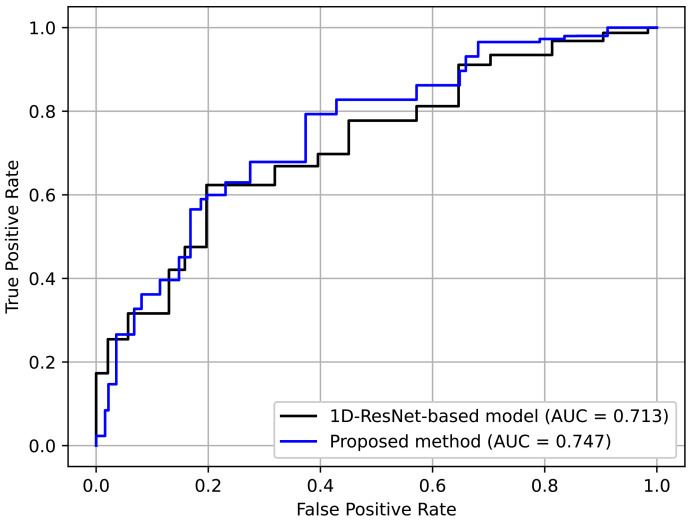
ROC curves for non-RP-based method and ours.

Table 6 shows the results. Here, it can be seen that the proposed method achieved better perfomance. This indicates the effectiveness of using an RP-based format, which preserves complex temporal patterns despite its simplicity. Figure 16 depicts the ROC curves for comparison. Again, it can be seen that the curve for the proposed method was generally better.

### 4.7. Ablation Study

In this section, we present an ablation study of the various techniques proposed. We tested whether to use the augmentation transform or not (“Transform”), as well as whether to use the additional protocol losses or not (“Using all labels”) as in (2). We also tested two other alternatives for selective dropout, i.e., “Dropout” and “Weight concat”. “Dropout” refers to a case where a plain dropout was used instead of the selective dropout. The dropout probability was set to 0.5. “Weight concat” refers to a method in which no form of dropout was used; instead, different parts of the final concatenated feature vector were simply weighted according to the probability values of selective dropout. This can be interpreted in another way, i.e., it is the selective dropout operating in ‘evaluation mode’ even during the training phase. These two alternatives were introduced to demonstrate the effectiveness of selective dropout.

Comparing all the techniques in Table 7, we can confirm that the selective dropout had the best impact on performance and was better than other similar techniques, i.e., dropout and weight concat. Moreover, we confirmed that adding augmentation transform and using the additional losses had a relative minor but meaningful impact on the performance.

**Figure 17:**
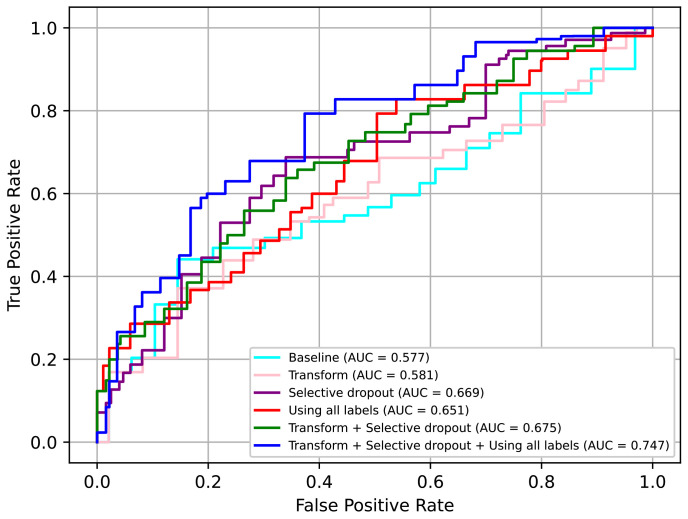
ROC curves for various models in ablation study.

In conclusion, we can confirm that all the proposed techniques, i.e., the augmentation transform, selective dropout, and use of all labels including individual protocol labels, had significant positive effects on performance. In particular, when using all three techniques, the AUC value increased by approximately 0.17 points from the baseline. The ROC curves for some of the above models are presented in Figure 17. Here, it can be seen that using all three techniques was clearly advantageous.

## 5. Conclusions

We proposed a deep-learning-based screening method for patients with stroke using neurological examination videos. Based on experimentation, using ResNet-18 architecture with overlapped N-divided RP pretrained with protocol tests, and applying the three additional techniques was the most effective, with an AUC value of 0.747. The proposed method has many potential practical applications, such as remote screening using a kiosk device or a mobile device.

In the experiments, we found that reducing the importance of the subnetworks for facial expression protocols using selective dropout was able to improve performance. We conjecture that the main reason for this was the biased facial landmark detector. However, including these protocols as a part of the input is still meaningful for the final diagnosis.

The KUAH dataset used in this study had some limitations, including its size and data distribution. Accordingly, the generality of the proposed method must be investigated further based on larger datasets. Future studies will extend the proposed framework to other types of neurological disorders, such as Parkinson’s disease. The use of multi-modal features, such as voice data or thermal images, which also contain important symptoms of stroke, represents another promising direction.

## Figures and Tables

**Table 1:** Results of pre-trained subnetworks on neurological protocols (AUC).

EOM	Facial Palsy	Dysarthria/Aphasia	Weakness	Finger Tab	Ataxia	Gait
0.533	0.541	0.505	0.822	0.795	0.679	0.677

**Table 2:** Stroke screening results with different pre-training strategies.

Method	AUC
Random weights	0.556
Pre-trained with NTU-RGB+D dataset	0.696
Pre-trained with protocols	0.747

**Table 3:** Stroke screening results with different learning rates in fine-tuning.

Method	$\mathrm{Lr}=10^{-3}$	$\mathrm{Lr}=10^{-4}$	Freeze
pre-trained with NTU-RGB+D dataset	0.671	0.696	0.658
pre-trained with protocols	0.719	0.747	0.713

**Table 4:** Stroke screening results with *N*-divided RP and overlapped *N*-divided RP.

Method	AUC
*N*-divided RP	0.724
overlapped *N*-divided RP	0.747

**Table 5:** Results with different network backbones.

	ResNet-18	ResNet-34	ResNeXt-50	EfficientNet-B2
AUC	0.747	0.703	0.682	0.728

**Table 6:** Comparison of RP- and non-RP-based methods.

Method	AUC
Proposed method	0.747
1D-ResNet-based model	0.713

**Table 7:** Results of ablation studies.

Transform	Dropout	Selective Dropout	Weight Concat	Using All Labels	AUC
					0.577
√					0.581
	√				0.579
		√			0.669
			√		0.644
				√	0.651
√		√			0.675
√			√	√	0.692
√		√		√	0.747

## Data Availability

Not applicable.

## Abbreviations

The following abbreviations are used in this manuscript:

AUC	Area Under the Curve
CNN	Convolutional Neural Network
CPSS	Cincinnati Pre-hospital Stroke Scale
EOM	Extra Ocular Movement
FAST	Face Arm Speech Test
IRB	Institutional Review Board
IQR	Inter Quartile Range
KUAH	Korea University Ansan Hospital
ML	Machine Learning
ROC	Receiver Operating Characteristic
RP	Recurrence Plot
